# Sex Differences in Presentation but Not in Outcome for ACTH-Dependent Cushing's Syndrome

**DOI:** 10.3389/fendo.2019.00580

**Published:** 2019-08-30

**Authors:** Leonie H. A. Broersen, Femke M. van Haalen, Tina Kienitz, Nienke R. Biermasz, Cristian J. Strasburger, Olaf M. Dekkers, Alberto M. Pereira

**Affiliations:** ^1^Division of Endocrinology, Department of Medicine, Leiden University Medical Center, Leiden, Netherlands; ^2^Center for Endocrine Tumors Leiden (CETL), Leiden University Medical Center, Leiden, Netherlands; ^3^Department of Endocrinology, Diabetes and Nutrition, Charité Universitätsmedizin Berlin, Corporate Member of Freie Universität Berlin, Humboldt-Universität zu Berlin, Berlin Institute of Health, Berlin, Germany; ^4^Department of Clinical Epidemiology, Leiden University Medical Center, Leiden, Netherlands

**Keywords:** Cushing's disease, ectopic Cushing's syndrome, sex differences, clinical picture, clinical outcome

## Abstract

**Background:** Sex differences in clinical picture of ACTH-dependent Cushing's syndrome are controversial, except for the known higher prevalence in females. We compared a broad range of potential differences to enable a more accurate understanding of the clinical picture of sex-specific ACTH-dependent Cushing's syndrome.

**Methods:** Cohort study including consecutive patients with ACTH-dependent Cushing's syndrome from Leiden and Berlin diagnosed between 2000 and 2016. We compared clinical presentation, biochemical parameters, diagnostic tests, surgical outcome, and comorbidities between men and women.

**Results:** We included 130 patients: 37 males and 93 females. With similar cortisol concentrations, ACTH concentrations were higher in males than females at time of diagnosis (median: 116 vs. 57 ng/L). The prevalence of osteoporosis was higher in males than in females (48.6 vs. 25.0%), persisting after surgery, with more vertebral fractures (16.2 vs. 5.4%) before surgery. Males showed more anemia (75.9 vs. 36.8%) after surgery. There were no differences in etiology, pituitary tumor size, diagnostic and therapeutic strategy, or surgical outcome between sexes.

**Conclusions:** Based on this study, males and females with ACTH-dependent Cushing's syndrome present different clinical patterns. However, these differences do not justify different diagnostic strategies or treatment based on sex, considering the similar surgical outcome. Clinicians should be alert to diagnose accompanying osteoporosis (with fractures) in male patients with ACTH-dependent Cushing's syndrome.

## Introduction

Cushing's syndrome is characterized by endogenous glucocorticoid excess, either adrenocorticotropic hormone (ACTH)-dependent or ACTH-independent, both with a variety of underlying causes (1). The vast majority of patients has Cushing's disease caused by an ACTH-secreting pituitary adenoma, with an estimated incidence of 1.2–1.7 per million each year (2). First-choice treatment for Cushing's disease is transsphenoidal pituitary surgery, selectively removing the corticotroph adenoma (3). Ectopic Cushing's syndrome is a rare condition resulting from a non-pituitary ACTH-producing source, and is generally approached by removing the ACTH-producing tumor, if identified and resectable. Excess of glucocorticoids causes osteoporosis, central obesity, insulin resistance, dyslipidemia, hypertension, hypercoagulability, and neuropsychiatric disorders (4, 5). Despite biochemical cure, mortality risk remains increased in patients with Cushing's disease (6).

Sex distribution differs markedly between different etiologies. There is consensus that Cushing's disease occurs up to five times more often in females, and that male patients with ACTH-dependent Cushing's syndrome have a relatively higher risk of an ectopic ACTH-secreting tumor. However, exact figures are lacking and a pathophysiological explanation for the sex distribution is absent (7, 8). Interestingly, the female preponderance is not yet present in prepubertal cases, suggesting that males are diagnosed with Cushing's syndrome at a younger age (9, 10). This is confirmed by some studies in adults with Cushing's syndrome (11, 12), but rejected by others (13, 14). Some cohort studies have reported males to have more severe clinical presentation (higher body mass index and waist circumference, reduced libido and sexual dysfunction, more striae, myopathy, and hypokalemia), biochemical parameters (higher ACTH, serum cortisol, and urinary free cortisol [UFC] concentrations), complications at diagnosis (higher HbA1c concentrations, more often hypertension, anemia, spine osteoporosis with vertebral fractures, rib fractures, and hypercoagulable state), and worse outcome after surgery (more often anemia, lower cortisol normalization rate, and higher recurrence rate), than females (8, 9, 11–16). Pituitary tumors were less easily visualized by pituitary magnetic resonance imaging (MRI) in males (12, 13). One study reported more macroadenomas, with higher invasion and apoplexy rate (11), whereas another study reported equal percentages of macroadenomas in males and females (12). No differences were found in quality of life between males and females on multiple validated questionnaires (CushingQoL, EQ-VAS, and SCL-90-R) (8, 11, 17). Table 1 summarizes the reported literature regarding sex differences (also reported as gender differences) in Cushing's syndrome (8–18).

**Table 1:** Studies on sex differences in Cushing's syndrome.

**References**	**Study center, country**	**Study type**	**Number of patients (male/female)**	**Type of patients**	**Mean age (years)**	**Study outcome: sex differences**
**STUDIES WITH SEX DIFFERENCES AS PRIMARY STUDY AIM**						
Ambrogio et al. (16)	Milan, Italy	Cohort	80 (17/63)	Cushing's disease	39.1 (range: 15–62)	Male: lower mean hemoglobin and RBC values than healthy subjects, with increased MCV. RBC and hemoglobin correlated to testosterone levels. After surgery more anemic, slower normalization of hemoglobin. Both sexes: hemoglobin reduced 1.5-2x after surgery, regardless of surgical outcome. MCV decreased 3 months after surgery.
Huan et al. (11)	Ji'nan, China	Cohort	87 (23/64)	Cushing's disease	43.33 (range: 23–65)	Male: younger age at diagnosis, larger adenoma diameter, higher invasion rate and apoplexy rate, more osteoporosis, hypokalemia, sexual dysfunction, and hypertension, higher preoperative and postoperative (6 months after surgery) cortisol levels, and a higher recurrence rate (30.4 vs. 7.8%). Both sexes: no differences in CushingQoL scores. Cortisol values 3 days postoperatively lower than before and 6 months after surgery.
Libuit et al. (9)	Bethesda, USA	Cohort	102 (54/48)	Cushing's disease, only pediatric patients	12.9 (SD: 3.0)	Male: more likely to present with higher BMI Z-score, lower height Z-score, higher plasma ACTH. Female: no specific signs or symptoms more often at presentation, increased risk of metabolic syndrome based on LDL at presentation. Both sexes: no difference in cure rate, in all patients decrease in prevalence of metabolic syndrome after surgery, equal tumor size.
Liu et al. (14)	Shanghai, China	Cohort	73 (13/60)	Cushing's disease	Male: 30.0 (range: 14–64) Female: 33.5 (range: 16–62)	Male: significantly higher ACTH (not explained by tumor size), BMI, HbA1c, ALT, AST, GGT, systolic blood pressure, and hemoglobin, more frequently purple striae, more fatty liver assessed by ultrasound. Both sexes: no differences in plasma cortisol, no difference in age.
Milian et al. (17)	Tübingen, Germany	Cohort	72 (11/61)	Cushing's disease, only patients biochemically cured	45.9 (range: 22-76)	Male: prolonged time to diagnosis strong predictive factor for worse psychopathological status (multiple dimensions, SCL-90-R). Both sexes: no significant difference in the frequency of psychopathology (in any of the dimensions). Presence of hypocortisolism was associated with phobic anxiety in male and psychoticism in female patients. Hypopituitarism was correlated with somatization in male and psychoticism in female patients.
Pecori Giraldi et al. (12)	Milan, Italy	Cohort	280 (47/233)	Cushing's disease	Male: 30.5 (SEM: 1.93) Female: 37.0 (SEM: 0.86)	Male: presentation at younger age, higher UFC and ACTH, lower sensitivity of high dose dexamethasone test, more symptoms indicative of hypercatabolic state (osteoporosis, muscle wasting, striae, nephrolithiasis), more often negative pituitary imaging, and immediate and late surgical outcome less favorable (lower surgical success rate and more frequent recurrence). Both sexes: similar time interval between appearance of first symptoms of hypercortisolism and diagnosis. Same percentage macroadenoma.
Rockall et al. (18)	London, UK	Cohort	31 (7/24)	Cushing's disease (*n* = 20) adrenal Cushing's syndrome (*n* = 5) ectopic Cushing's syndrome (*n* = 3) unknown etiology (*n* = 3)	45 (range: 17-79)	Male: significant increase in the V:S (visceral fat:subcutaneous fat) ratio compared with non-cushingoid controls (control data from literature). Female: significant increase in the V:S ratio compared with non-cushingoid controls. Both sexes: There was no difference in the V:S ratio between male and female patients.
Storr et al. (10)	London, UK	Cohort	50 (21/29)	Cushing's disease, only patients 30 years of age or less	18.0 (range: 6.4–30.0)	In patients 18 years of age or younger, there was no difference in the severity of hypercortisolemia or ACTH at diagnosis between males and females.
Zilio et al. (13)	Padova, Italy	Cohort	84 (17/67)	Cushing's disease	42.2 (range: 15–70)	Male: higher UFC and ACTH values, lower ACTH response to DDAVP stimulation. Pituitary tumor less easily visualized by pituitary MRI. More frequent or more severe complications, in particular hypokalemia, hypercoagulable state, and osteoporosis at lumbar spine, with consequent higher risk of vertebral fractures. Male sex was an independent risk factor for dyslipidemia, severity of hypertension, lumbar osteoporosis and fractures. Both sexes: No differences in age at diagnosis, disease duration and BMI. The prevalence of hypogonadism did not significantly differ.
**STUDIES WITH SEX DIFFERENCES AS SECONDARY STUDY AIM**						
Patil et al. (15)	Stanford, USA	Cohort	3525 (649/2871)	Cushing's disease	Reported only in categories: <18: 147 18-44: 2246 45-64: 966 >64: 161	Women were less likely than men to have an adverse outcome (OR 0.3).
Valassi et al. (8)	Multicenter (Europe)	Cohort	481 (91/391)	Cushing's disease (*n* = 317). adrenal Cushing's syndrome (*n* = 130). ectopic Cushing's syndrome (*n* = 24). other etiology (*n* = 10)	44.2 (range: 15-84)	Male: significantly higher proportion ectopic Cushing's syndrome than other etiologies. Reduced libido more prevalent than in women. Higher prevalence of spine osteoporosis, and more vertebral and rib fractures. Mean waist significantly higher. Hypertension (83%), myopathy (71%), and reduced libido (69%) more common. Female: weight gain more common. Both sexes: no difference in the specialists consulted (before correct diagnosis) (other than gynecologists). Mean CushingQoL and EQ-VAS score not different.

*ACTH, adrenocorticotropic hormone; ALT, alanine aminotransferase; AST, aspartate aminotransferase; BMI, body mass index; CushingQoL, Cushing Quality of Life questionnaire; EQ-VAS, EuroQoL-visual analog scale; GGT, gamma-glutamyltransferase; HbA1c, glycated hemoglobin; LDL, low-density lipoprotein; MCV, mean corpuscular volume; OR, odds ratio; RBC, red blood cell; SCL-90-R, Symptom Checklist–revised; SD, standard deviation; SEM, standard error of the mean; UFC, urinary free cortisol*.

Potential differences between males and females could for example be based on: (1) Different concentrations of corticosteroid binding globulin (CBG) (19), (2) Different ACTH secretion and cortisol production rate (20, 21), (3) Different interaction between corticosteroids and the gonadotroph axis, and (4) Different disease entity of Cushing's syndrome. A large cohort study focusing on a broad range of potential differences between both sexes is needed to confirm the sometimes conflicting results from previous small studies focusing on a limited number of aspects, to enable a more accurate understanding of the clinical picture of sex-specific ACTH-dependent Cushing's syndrome.

### Study Aims

To compare the phenotype of male and female patients with ACTH-dependent Cushing's syndrome regarding: (1) Clinical presentation, (2) Biochemical parameters and diagnostic test results, (3) Surgical outcome (i.e., percentage remission, hydrocortisone dependency, recurrence, and mortality), and comorbidities. Based on previous literature, we hypothesized that males show more symptoms related to hypercortisolism at diagnosis, with higher concentrations of ACTH and cortisol, and more comorbidity at diagnosis, specifically hypertension, anemia, and osteoporosis. We also hypothesized that males show worse outcome after surgery regarding remission and recurrence rate.

## Methods

### Study Population

Consecutive patients with ACTH-dependent Cushing's syndrome from the Leiden University Medical Center and the Charité Universitätsmedizin Berlin were included. Only patients with a diagnosis from January 1st 2000 onwards were included, as this guaranteed data collection from equal time periods for both centers. There were no restrictions regarding treatment (transsphenoidal surgery, adrenalectomy, radiotherapy, medical treatment, and ectopic tumor resection).

The process to diagnose ACTH-dependent Cushing's syndrome was published previously (22). Cut-off levels for the used diagnostic tests varied between study center and time period, due to use of different assays. All patients had pituitary imaging by MRI, except for three patients who had computed tomography (CT) only: one Cushing's disease patient due to a contraindication for MRI, and two ectopic Cushing patients who already had CT for other reasons, revealing tumors with a high suspicion of ectopic ACTH secretion.

First choice treatment for Cushing's disease was transsphenoidal adenomectomy (TSA). Approximately half of the TSA surgeries was performed by microscopic surgery, and the other half was performed by endoscopic surgery. One patient underwent bilateral adrenalectomy as first treatment (ACTH-dependent Cushing's syndrome without clear pituitary adenoma, but also no ectopic source), two did not have surgery yet within the study period, and two were being treated long-term medically (one with cabergoline and one with levoketoconazole in a controlled trial setting). For ectopic Cushing's syndrome, first choice treatment was removal of the ectopic ACTH-producing tumor. In 2001, one patient had TSA first, as a pituitary adenoma was identified on MRI, before the diagnosis of ectopic Cushing's syndrome was established. Two patients underwent adrenalectomy as first treatment (one unilateral because the ectopic tumor was located in the adrenal, and one bilateral because this could be combined with a nephrectomy for a renal cell carcinoma), and two patients died before treatment was instituted. The method of postoperative evaluation and definitions of surgical outcomes (remission, recurrence, and persistent disease) were presented previously (22).

### Outcomes and Follow-Up

Outcomes of interest were clinical presentation of ACTH-dependent Cushing's syndrome, surgical outcome, and short- and long-term morbidity. Surgical outcome included percentage remission, hydrocortisone dependency, recurrence, and mortality. Hydrocortisone dependency was measured 3 months after surgery and was divided in three categories: (a) Absolute deficiency if insufficient cortisol response to a stimulation test [corticotropin-releasing hormone (CRH)-test, ACTH-test or insulin tolerance test], (b) Hydrocortisone for symptoms despite normal cortisol response to stimulation, and (c) Pragmatic hydrocortisone replacement (without stimulation test). For diagnosing post-operative adrenal insufficiency, the CRH test was usually performed in Leiden, whereas the ACTH test was usually performed in Berlin. Neither of the study centers usually performed the insulin tolerance test. For some patients, no stimulation test was performed due to low post-operative cortisol levels and requirement of hydrocortisone replacement therapy.

Short-term morbidity (≤3 months after first surgery) included (1) Anemia (defined as hemoglobin concentrations of <8.5 mmol/L for males and <7.5 mmol/L for females, measured within 2 weeks after surgery), (2) Anterior pituitary deficiency other than ACTH requiring medication (number of deficient axes, with gonadal axis deficiency also described separately), (3) Severe bleeding (requiring surgical intervention or bleeding described as severe in patient file), and (4) Cardiovascular event (thrombosis, pulmonary embolism, cerebrovascular accident, transient ischemic attack, and myocardial ischemia).

Long-term morbidity (>3 months after first surgery) included (1) Anterior pituitary deficiency (one year after surgery), (2) Hypertension (*de novo* as well as persisting after surgery), (3) Diabetes mellitus (*de novo* as well as persisting after surgery), (4) Neuropsychiatric morbidity (complaints as well as consultation of psychologist or psychiatrist), (5) Osteoporosis (defined as a bone mineral density T-score of −2.5 standard deviation [SD]), and (6) Fractures (symptomatic as well as radiologically diagnosed asymptomatic fractures were included, clinical vertebral and femoral fractures described separately). Anterior pituitary deficiency was described only for patients after a transsphenoidal adenomectomy.

We followed patients from date of diagnosis until death, loss to follow-up, or 31 December 2016, whichever came first. The following patient information was collected at time of diagnosis: age, comorbidities (cardiovascular event, hypertension, diabetes mellitus, dyslipidemia, neuropsychiatric morbidity, anemia, osteoporosis, fractures in patient history), and all eight items of the Cushing's syndrome Severity Index score (CSI score) (23).

Ectopic Cushing's syndrome was classified according to the following underlying disorders: neuroendocrine tumor of the gastrointestinal tract, lung tumor, and other source of ACTH production. Pituitary tumor size was divided into microadenomas (≤10 mm) and macroadenomas (>10 mm).

### Risk of Bias

This study included all eligible patients to prevent selection bias. However, selective loss to follow-up could have led to selection bias, if more patients from one sex were lost to follow-up than from the other sex caused by e.g., presence of comorbidities. This could alter the percentages of patients with long-term comorbidity after treatment in our study, leading to biased results. Confounding was not assessed as a potential source of bias, as study groups were formed based on sex, and no factor of interest was thought to influence sex. Factors associated with sex could have influenced our results due to selection bias, e.g., by differences in age, and these factors were compared between both sexes, as described in the next paragraph.

### Statistical Analysis

The following contingency tables were prepared, comparing male to female patients with ACTH-dependent Cushing's syndrome: (1) Demographic characteristics, phenotype of Cushing's syndrome, and medical history (above mentioned patient information collected at time of diagnosis, as well as duration of follow-up), and (2) Surgical outcome, and short- and long-term morbidity. Adjuvant treatments, including all treatments other than the primary treatment for Cushing's syndrome, were reported. Furthermore, diagnostic strategy and results (biochemical parameters at diagnosis, type and result of radiologic imaging, simultaneous bilateral inferior petrosal sinus sampling, etiology of Cushing's syndrome, tumor size for pituitary adenomas, medical treatment prior to surgery, histology results, and immunohistochemistry results) were compared between male and female patients. The unpaired *T*-test was used to compare outcomes for continuous variables, and for categorical variables the two-sample test of proportions was used. To correct for multiple testing, the Bonferroni method was used and tests were considered significant if *p* < 0.001, although this was probably too conservative for this study due to correlations between the analyses (e.g., osteoporosis and fractures). All performed analyses were reported in this article. In the tables, percentages were reported according to the total number of patients with a valid value for the specific parameter. If per parameter, data were missing for ≥5% of patients, this was marked in the tables. If variables with ≥5% missing data showed a clear difference between sexes, we also calculated percentages according to total number of patients, thereby assuming that patients with a missing value were rightfully unmeasured, and reported this in the results section only.

Kaplan-Meier curves were constructed for overall survival since time of diagnosis, and for recurrence-free survival since time of surgery between male and female patients. For recurrence-free survival, only patients at risk for recurrence were included in the analysis. Cox proportional hazard regression analyses were performed to provide hazard ratios with 95% confidence intervals.

IBM SPSS Statistics 23.0 (IBM Corp, Armonk, NY, USA) was used to perform all statistical analyses, except the two-sample test of proportions (command: prtesti), which was performed using Stata 14.2 (Stata Corp., College Station, TX, USA), to calculate the difference between two proportions with 95% confidence interval, as this was not provided by SPSS. Patients gave written informed consent for use of their data for scientific research in accordance with the Declaration of Helsinki. Permission from the ethical committees in the LUMC and Charité Universitätsmedizin was granted. The Strengthening the Reporting of Observational Studies in Epidemiology (STROBE) guidelines were used for reporting (24).

## Results

### Study Population (Table 2)

In total, 130 patients were included (*n* = 85 from Leiden, *n* = 45 from Berlin), of whom 37 are males (28.5% of total, mean age: 45.3 years, range: 14–74 years) and 93 females (mean age: 44.8 years, range: 10–80 years). With similar serum cortisol concentrations (median: 670 vs. 680 nmol/L) and UFC (median: 4.5 times upper limit of normal [ULN] vs. 3.4 times ULN), males had higher ACTH concentrations at time of diagnosis when compared to females (median: 116 vs. 57 ng/L). There were no differences regarding etiology of Cushing's syndrome (males 5.4% ectopic, females 4.3% ectopic), and tumor size of pituitary adenoma (males 40.0% macroadenomas, females 32.2% macroadenomas). Radiologic findings (pituitary adenoma, ectopic ACTH-secreting tumor, no tumor, or inconclusive), and percentage of patients with inferior petrosal sinus sampling were comparable for both sexes. Osteoporosis was more prevalent at time of diagnosis in males (48.6 vs. 25.0%) than in females. Male patients also reported more fractures in their patient history (27.0 vs. 19.6%), mainly vertebral (16.2 vs. 5.4%). None of these differences was statistically significant after Bonferroni correction. Males less often reported sex-related disturbances than females (positive item on Cushing's syndrome Severity Index score: 22.8 vs. 64.0%).

**Table 2:** Demographic characteristics and phenotype of Cushing's syndrome.

	**Male**		**Female**		Tested difference (95% CI; *p*-value*)
Total number of patients (*N*, %)	37	100.0	93	100.0	
Age at diagnosis in years (mean, SD)	45.3	16.4	44.8	15.1	0.5 (−5.4 to 6.5; *p* = 0.87)
Duration of follow-up in years (median, IQR)	5.9	2.2-9.8	5.6	1.9–12.2	0.6 (−1.2 to 2.5; *p* = 0.50)
**Comorbidities at diagnosis**					
- Cardiovascular event (*N*, %)	6	16.2	10	10.9	5.3% (−8.2 to 18.8%; *p* = 0.41)
- Hypertension (*N*, %)	28	75.7	64	69.6	6.1% (−10.6 to 22.8%; *p* = 0.49)
- Diabetes mellitus (*N*, %)	12	32.4	28	30.4	2.0% (−15.8 to 19.8%; *p* = 0.82)
- Dyslipidemia (*N*, %)	7	18.9	18	19.6	0.7% (−14.3 to 15.7%; p = 0.93)
- Neuropsychiatric morbidity (*N*, %)	14	37.8	37	39.8	2.0% (−16.5 to 20.5%; *p* = 0.83)
- Patients with anemia (*N*, %)°	3	9.1	7	8.4	0.7% (−10.8 to 12.2%; *p* = 0.90)
Hemoglobin concentration – average for all patients in mmol/L (mean, SD)°	9.4	1.0	8.8	1.0	
- Osteoporosis (*N*, %)	18	48.6	23	25.0	23.6% (5.2 to 42.0%; *p* = 0.009)
- Fractures in patient history (*N*, %)	10	27.0	18	19.6	7.4% (−9.0 to 23.8%; *p* = 0.36)
Vertebral fracture (*N*, %)	6	16.2	5	5.4	
Femoral fracture (*N*, %)	1	2.7	1	1.1	
**Cushing's syndrome Severity Index score (mean, SD)**	6.2	3.1	6.9	2.6	−0.7 (−1.8 to 0.4; *p* = 0.20)
- Fat distribution (*N*, %)	31	88.6	86	96.6	8.0% (−3.2 to 19.2%; *p* = 0.083)
Mild (*N*, %)	16	45.7	28	31.4	
Severe (*N*, %)	15	42.9	58	65.2	
- Skin lesions (*N*, %)	27	77.1	64	71.9	5.2% (−11.6 to 22.0%; *p* = 0.56)
Mild (*N*, %)	20	57.1	40	44.9	
Severe (*N*, %)	7	20.0	24	27.0	
- Muscle weakness (*N*, %)	21	60.0	51	57.3	2.7% (−16.5 to 21.9%; *p* = 0.78)
Mild (*N*, %)	8	22.9	19	21.3	
Severe (*N*, %)	13	37.1	32	36.0	
- Mood disorder (*N*, %)	14	40.0	42	47.2	7.2 (−12.1 to 26.5%; *p* = 0.47)
Mild (*N*, %)	9	25.7	24	27.0	
Severe (*N*, %)	5	14.3	18	20.2	
- Hypertension (*N*, %)	28	80.0	63	70.8	9.2% (−7.1 to 25.5%; *p* = 0.30)
Mild (*N*, %)	24	68.6	51	57.3	
Severe (*N*, %)	4	11.4	12	13.5	
- Diabetes mellitus (*N*, %)	11	31.4	32	36.0	4.6% (−13.7 to 22.9%; *p* = 0.63)
Mild (*N*, %)	3	8.6	16	18.0	
Severe (*N*, %)	8	22.8	16	18.0	
- Hypokalemia (*N*, %)	11	31.4	18	20.2	11.2% (−6.3 to 28.7%; *p* = 0.19)
Mild (*N*, %)	3	8.6	5	5.6	
Severe (*N*, %)	8	22.8	13	14.6	
- Sex-related disturbances (*N*, %)	8	22.8	57	64.0	41.2% (24.1 to 58.3%; *p* = 0.000)
Mild (*N*, %)	2	5.7	29	32.6	
Severe (*N*, %)	6	17.1	28	31.4	

CI, confidence interval; IQR, interquartile range; SD, standard deviation.

* Due to the Bonferroni correction, tests were considered significant if p < 0.001,

° *data were missing for ≥5% of patients*.

Males and females were treated for Cushing's syndrome similarly: percentage of patients with cortisol-lowering medication preoperatively (males 68.6%, females 65.9%) was comparable. After surgery, histology results (tumor identified, no tumor identified, or inconclusive) and immunohistochemistry results (ACTH-positive or negative) were also similar for both sexes.

**Figure 1:**
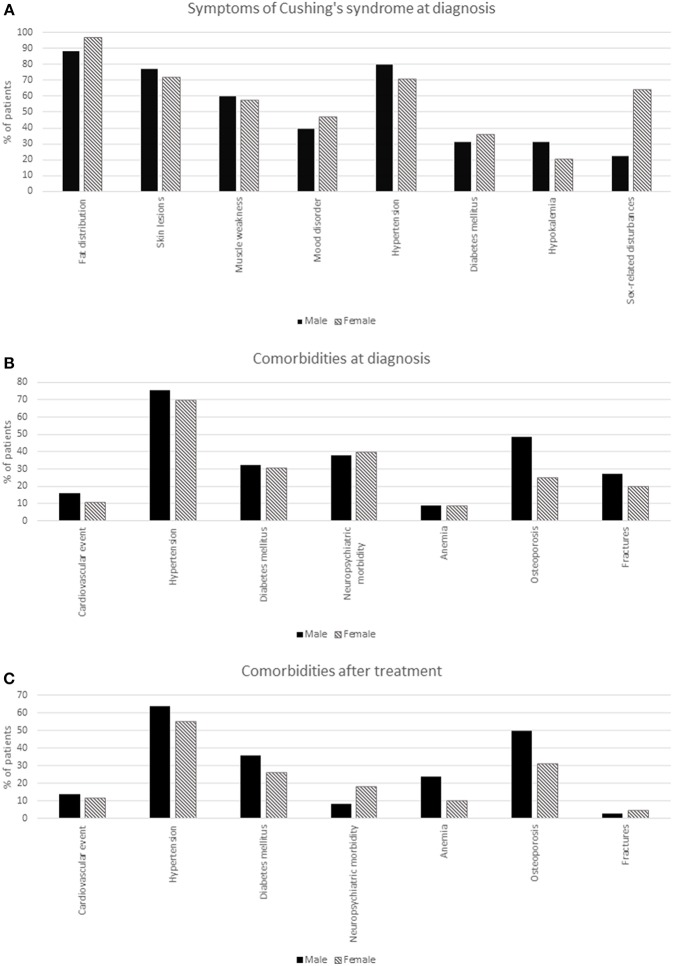
Comorbidities and symptoms of ACTH-dependent Cushing's syndrome by sex. **(A)** Symptoms of Cushing's syndrome at diagnosis. **(B)** Comorbidities at diagnosis. **(C)** Comorbidities after treatment.

Twenty patients were lost to follow-up [four males (10.8%) and sixteen females (17.2%), seventeen patients from Berlin, three patients from Leiden] after an average follow-up time of 71 months. For a detailed description of demographic characteristics, see Table 2, and Figures 1A–C. As there were only six patients with ectopic Cushing's syndrome, no analyses were performed stratified by etiology. The ectopic ACTH-secreting tumors were: pancreatic neuroendocrine tumor (*n* = 1), lung tumors [carcinoid (*n* = 1) and carcinoma (*n* = 1)], thymoma (*n* = 1), thymic carcinoma (*n* = 1), and pheochromocytoma (*n* = 1).

### Surgical Outcome

**Figure 2:**
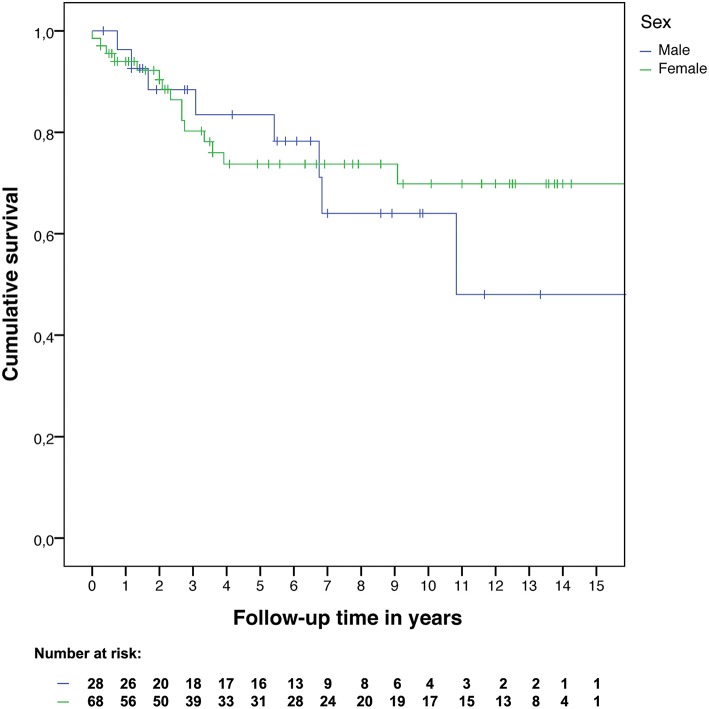
Recurrence-free survival by sex.

There were 34 male patients with at least 3 months follow-up after surgery, of whom 28 were in remission (82.4%), and 22 were hydrocortisone dependent (64.7%). Of the 28 males in remission after surgery, recurrence occurred in eight patients (28.6%), after a mean of 55 months (range: 9–130 months). Five of these eight patients were hydrocortisone dependent 3 months after first surgery (62.5%). There were 85 female patients with at least 3 months follow-up after surgery, of whom 68 were in remission (80.0%), and of whom 55 were hydrocortisone dependent (64.7%). Of the 68 females in remission after surgery, recurrence occurred in 15 patients (22.1%), after a mean of 30 months (range: 0–109 months). Eight of these 15 patients were hydrocortisone dependent 3 months after first surgery (53.3%). The hazard ratio for recurrence was 1.22 (95% confidence interval: 0.52–2.89) for males compared to females (Figure 2). Three months after surgery, ACTH concentrations were higher in males than in females (mean: 30 vs. 11 ng/L). More detailed surgical outcome results can be found in Table 3.

**Table 3:** Surgical outcome and short and long-term morbidity.

	**Male**		**Female**		Tested difference (95% CI; *p*-value*)
**SURGICAL OUTCOME**					
- Cortisol direct postoperatively in nmol/L (median, IQR)°	220	50–510	60	30–360	100 (−20 to 220; *p* = 0.096)
- Cortisol 3 to 6 months postoperatively in nmol/L (median, IQR)°	180	80–280	130	40-320	30 (−70 to 120; *p* = 0.56)
- ACTH 3 to 6 months postoperatively in ng/L (median, IQR)°	30	8-72	11	5-33	50 (−13 to 113; *p* = 0.12)
- Hydrocortisone dependency (*N*, %)	22	66.7	55	67.1	0.4% (−18.6 to 19.4%; *p* = 0.97)
- Absolute deficiency (*N*, %)	19	57.6	51	62.2	
- Normal cortisol response (*N*, %)^#^	0	0.0	1	1.2	
- Pragmatic replacement (*N*, %)^§^	3	9.1	3	3.7	
- Persistent disease (*N*, %)	6	17.1	17	19.5	2.4% (−12.6 to 17.4%; *p* = 0.76)
- Recurrent disease (*N*, %)	8	22.9	15	17.2	5.7% (−10.3 to 21.7%; *p* = 0.47)
- Adjuvant treatment (*N*, %)	15	41.7	32	36.4	5.3% (−13.7 to 24.3%; *p* = 0.58)
- Radiotherapy (*N*, %)	12	33.3	13	14.8	
- TSA (*N*, %)	8	22.2	22	25.0	
- Adrenalectomy (*N*, %)	2	5.6	6	6.8	
- Ectopic tumor resection (*N*, %)	0	0.0	1	1.1	
- Medical treatment (*N*, %)	5	13.9	10	11.4	
**SHORT TERM MORBIDITY:** **≤3 MONTH AFTER FIRST SURGERY**					
- Number of patients with anemia (*N*, %)°	22	75.9	25	36.8	39.1% (19.8 to 58.4%; *p* = 0.000)
Hemoglobin concentration – average for all patients in mmol/L (mean, SD)°	7.7	1.3	7.7	1.0	
- Anterior pituitary deficiency (*N*, %)^°+^	10	34.5	17	22.1	12.4% (−7.2 to 32.0%; *p* = 0.19)
- One axis (*N*, %)	6	20.7	13	16.9	
- Two axes (*N*, %)	2	6.9	3	3.9	
- Three axes (*N*, %)	2	6.9	1	1.3	
- Gonadal axis deficiency (*N*, %)	7	24.1	5	6.5	
- Bleeding, severe (*N*, %)	2	6.1	1	1.2	4.9% (−3.6 to 13.4%; *p* = 0.13)
-Cardiovascular event (*N*, %)	3	9.1	4	4.8	4.3% (−6.5 to 15.1%; *p* = 0.38)
**LONG TERM MORBIDITY:** **>3 MONTHS AFTER FIRST SURGERY**					
- Anterior pituitary deficiency after 1 year (*N*, %)^°+^	9	32.1	18	25.3	6.8% (−13.2 to 26.8%; *p* = 0.49)
- One axis (*N*, %)	4	14.3	14	19.7	
- Two axes (*N*, %)	3	10.7	2	2.8	
- Three axes (*N*, %)	2	7.1	2	2.8	
- Gonadal axis deficiency (*N*, %)	6	21.4	4	5.6	
- Hypertension (*N*, %)	23	63.9	48	55.2	8.7% (−10.2 to 27.6%; *p* = 0.37)
- Diabetes mellitus (*N*, %)	13	36.1	23	26.4	9.7% (−8.5 to 27.9%; *p* = 0.28)
- Neuropsychiatric morbidity (*N*, %)	3	8.3	16	18.2	9.9% (−2.2 to 22.0%; *p* = 0.17)
- Osteoporosis (*N*, %)	18	50.0	27	31.0	19.0% (0.0 to 38.0%; *p* = 0.047)
- Fractures (*N*, %)	1	2.8	4	4.6	1.8% (−5.2 to 8.8%; *p* = 0.65)
- Clinical vertebral fracture (*N*, %)	1	2.8	2	2.3	
- Femoral fracture (*N*, %)	0	0.0	0	0.0	

ACTH, adrenocorticotropic hormone; CI, confidence interval; IQR, interquartile range; SD, standard deviation; TSA, transsphenoidal adenomectomy.

* Due to the Bonferroni correction, tests were considered significant if p < 0.001,

° data were missing for ≥5% of patients,

+ only for patients with TSA.

# Hydrocortisone for symptoms despite normal cortisol response to CRH stimulation.

§ *Pragmatic hydrocortisone replacement (without stimulation test)*.

### Overall Survival

**Figure 3:**
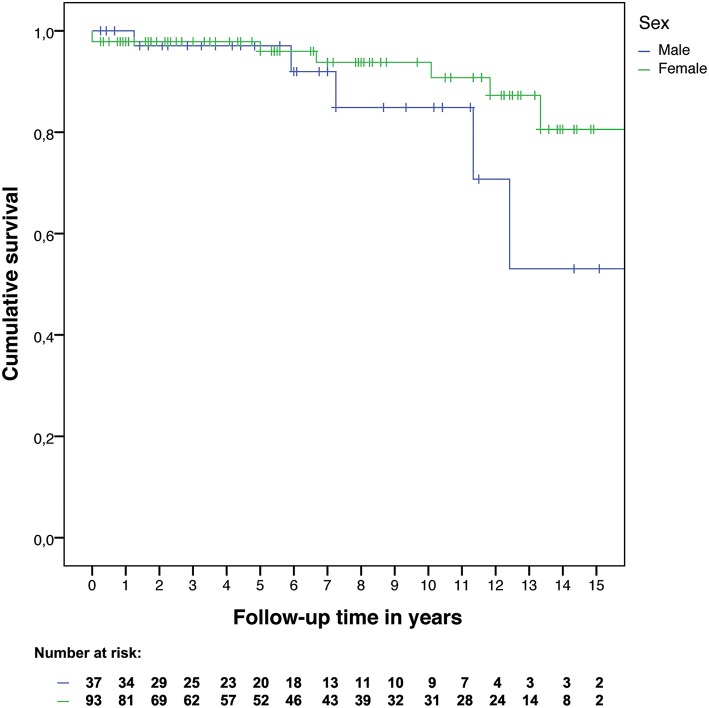
Overall survival by sex.

Within the study period 12 patients died, five males and seven females. The hazard ratio for mortality was 2.35 (95% confidence interval: 0.73–7.51) for males compared to females. The Kaplan-Meier curves of overall survival are shown in Figure 3.

### Short- and Long-Term Morbidity

Postoperatively, anemia was more prevalent in males than in females (75.9 vs. 36.8% for patients with a valid value only). If all patients were included in this analysis, assuming that patients without a valid value were rightfully unmeasured, this difference was even larger (78.4 vs. 26.9%). After surgery, males continued to have osteoporosis more often than females (50.0 vs. 31.0%). Overall, anterior pituitary deficiency was more prevalent in males than females (34.5 vs. 22.1) 3 months after surgery. Specifically, gonadal axis deficiency needing replacement therapy was more prevalent in males (24.1 vs. 6.5%). This difference remained 1 year after surgery. More detailed morbidity results can be found in Table 3, and in Figures 1B,C.

## Discussion

In this cohort study, we compared clinical presentation, biochemical parameters, diagnostic test results, surgical outcome, and morbidity in male vs. female patients with ACTH-dependent Cushing's syndrome. With similar serum cortisol concentrations and UFC, males had higher ACTH concentrations at time of diagnosis than females, with no difference in etiology of Cushing's syndrome and pituitary tumor size between the sexes. Cushing's disease was the most common cause of ACTH-dependent Cushing's syndrome in both male and female patients. At diagnosis of Cushing's syndrome, we found a higher prevalence of osteoporosis with (vertebral) fractures in males than females. After surgery, the higher prevalence of osteoporosis with (vertebral) fractures persisted, and a higher prevalence of anemia in males than in females was found. It is important to note that there were no differences in surgical outcome, recurrence, or mortality between sexes. Thus, male patients with ACTH-dependent Cushing's syndrome seem to constitute a different clinical pattern regarding symptoms and biochemistry, which does, however, not affect the further diagnostic strategy, therapy, or surgical outcome in this cohort study, although comorbidities did differ between sexes. Therefore, no different diagnostic or therapeutic strategy is indicated based on sex. However, based on the differences in comorbidities, extra attention should be given to male patients for diagnosing and treating osteoporosis (with fractures).

A major strength of this study is that it includes a large cohort from two centers and focuses on a broad spectrum of potential differences between sexes, thereby allowing a more accurate description and clear understanding of the clinical picture of ACTH-dependent Cushing's syndrome based on sex, than previous cohort studies. The differences in biochemistry and morbidity found in this study are largely in line with previous studies: higher plasma ACTH (9, 12–14), more often anemia (16) and osteoporosis (with fractures) (8, 11–13). The lack of difference in etiology and pituitary tumor size was in agreement with some studies (9, 12), but not with others (8, 11). Similarly, the lack of difference in surgical outcome was in agreement with one study (9), but not with two others (11, 12). Thus, this study adds evidence to the existence of a sex difference with respect to ACTH, anemia, and osteoporosis (with fractures), and to the lack of a sex difference in etiology and pituitary tumor size. Due to the low number of fractures found in our study, no extensive analyses could be performed to compare the incidence of various types of fractures between men and women. We suggest future studies to focus on the occurrence of fractures in general in men and women with ACTH-dependent Cushing's syndrome, as well as vertebral and femoral fractures separately. The existence of a sex difference regarding surgical outcome is doubtful, as now two studies show a difference, whereas two others do not, indicating that more research is needed into the existence of a sex difference in surgical outcome. As the associations in our study are restricted to ACTH-dependent Cushing's syndrome as exposure, and not as outcome, no explanation can be provided as to why Cushing's disease is more prevalent in women than in men. This question could be answered by future studies examining potential causes of Cushing's disease.

When interpreting the results, the following study limitations should be taken into account. As this study compared a broad spectrum of potential differences between sexes, the Bonferroni method was used to control for multiple testing, leading to tests considered significant only if *p* < 0.001. Consequently, the cohort size was insufficient to detect any differences that were smaller than 40% between the study groups. In order to find a 20% difference between the study groups with a significance level of *p* < 0.001, we would have needed at least 450 patients in total, given the sex distribution in this study, which would have taken many more study centers or multiple extra decades to assemble. However, none of the previously published studies on potential sex differences controlled for multiple testing, without exception using a significance level of *p* < 0.05 despite using many statistical tests per study. This should be taken into account when comparing our study results to the literature. Furthermore, as there were only six patients with ectopic Cushing's syndrome, no analyses could be performed for patients with ectopic Cushing's syndrome separately.

Loss to follow-up could have led to selection bias, since more female than male patients were lost to follow-up. However, as no patient was lost to follow-up within 6 months postoperatively, and most study endpoints were measured within 6 months postoperatively, selective loss to follow-up is unlikely to have influenced study results.

As this study was performed retrospectively, using information from patient files, information could not always be collected from similar time points for each patient, leading to missing data in some patients (e.g., anemia at diagnosis), and information bias is likely to have occurred due to selective questioning of patients (e.g., sex-related disturbances in the Cushing's syndrome Severity Index score). This presumably led to the improbable low percentage of males reporting sex-related disturbances, whereas other studies found males to have sex-related disturbances more often than females (8, 11). Thus, it is important to inquire patients at disease presentation thoroughly about this subject and ideally also interview the patient's partner in this regard.

As should be discussed according to the STROBE guideline (24), this study is theoretically generalizable to all patients with ACTH-dependent Cushing's syndrome. However, the small number of patients with ectopic Cushing's syndrome precludes generalizability for this etiological ultrarare subgroup.

ACTH has been reported to be higher in males than in females with ACTH-dependent Cushing's syndrome consistently across multiple studies (9, 12–14). Some studies, that also reported higher concentrations of UFC in males than in females, suggested that ACTH-dependent Cushing's syndrome is a more aggressive disease in males (12, 13). The increased ACTH concentrations could not be related to larger tumor size of the pituitary adenoma (14). Likewise, as the percentage of patients with ectopic Cushing's syndrome was similar for both sexes, etiological differences are no satisfying explanation for the observed variation in ACTH concentrations. Until now, no study has found a convincing pathophysiological explanation why ACTH is higher in males than in females. However, also in healthy humans, ACTH secretion and cortisol production rate have been found increased in males compared with females (20, 21). Interestingly, higher concentrations of ACTH in males do not consistently seem to increase concentrations of cortisol (serum cortisol as well as UFC). As higher cortisol concentrations are assumed to represent aggressiveness of disease, the lack of increased cortisol concentrations reduces the probability that ACTH-dependent Cushing's syndrome in males actually is a more aggressive disease than in females. Future research with the aim to discover why ACTH is higher in males than in females could focus first on which factors other than pituitary tumor size or etiology might be related to a higher ACTH per sex.

Osteoporosis (with fractures) and anemia were more prevalent in males than in females, which may be explained, at least in part, by patient delay during the diagnostic process, as males may have less pronounced symptoms than females in the earlier stages of the disease (i.e., menstrual disturbances), leading to a greater delay in diagnosis and therefore more, and more severe complications at time of diagnosis in males. Furthermore, it has been suggested that interaction between corticosteroids and the gonadotropic axis leads to hypogonadism in males more often than in females, which may markedly influence bone damage in Cushing's syndrome (8, 13). This is in accordance with our study results, which showed postoperative hypogonadism more often in males than females. This hypogonadism may also cause an endocrine anemia due to the insufficient drive of testosterone on erythropoiesis (25). However, a meta-analysis of prior corticosteroid use and (osteoporotic) fracture risk found no difference between men and women (26). Increased fracture risk and decreased bone mineral density in hypercortisolism is mainly due to suppression of bone formation, which is negatively regulated by an osteocyte-produced factor: sclerostin. Sclerostin is a protein that acts as a key inhibitor of the Wnt signaling pathway in osteoblasts (27). Interestingly, in mice, glucocorticoids stimulate the production of sclerostin (28), whereas circulating sclerostin was decreased in the only available study in patients with Cushing's syndrome to date during hypercortisolism, and increased after correction of glucocorticoid excess (29). Differences in circulating sclerostin between the sexes were not reported in the limited number of patients studied. Both estradiol and testosterone regulate bone turnover, but circulating sclerostin appears to be differentially regulated by sex steroids in women and men (30). Specifically, estradiol, but not testosterone, prevented increases in sclerostin levels following induction of sex steroid deficiency in older men and women, indicating that changes in sclerostin production are likely affected by a complex interplay between estrogens and testosterone and glucocorticoids on bone turnover.

In conclusion, male patients with ACTH-dependent Cushing's syndrome seem to show a different clinical picture than females. However, no different diagnostic strategy or treatment is indicated based on sex, in view of the similar surgical outcome. Clinicians should pay more attention to male patients with ACTH-dependent Cushing's syndrome in particular regarding diagnosis and treatment of accompanying osteoporosis (with fractures).

## Data Availability

The datasets supporting the conclusions of this study are available upon reasonable request from the corresponding author.

## Ethics Statement

Patients gave written informed consent for use of their data for scientific research in accordance with the Declaration of Helsinki. Permission from the ethical committees in the LUMC and Charité Universitätsmedizin was granted.

## Author Contributions

LB, TK, NB, CS, OD, and AP contributed to the conception and design of the study. LB and FH collected the data and organized the database. LB performed the statistical analyses and wrote the first draft of the manuscript. All authors contributed to the manuscript revision, read and approved the submitted version.

### Conflict of Interest Statement

The authors declare that the research was conducted in the absence of any commercial or financial relationships that could be construed as a potential conflict of interest.
